# 高效液相色谱-紫外检测法同时测定洗面奶中4种*N*-月桂酰氨基酸表面活性剂

**DOI:** 10.3724/SP.J.1123.2023.09008

**Published:** 2024-01-08

**Authors:** Wenshan ZHUO, Jianfeng TANG, Jinsheng LIANG, Rihui CAO

**Affiliations:** 1.中山大学分析测试中心, 广东 广州 510275; 1. Instrumental Analysis and Research Center, Sun Yat-sen University, Guangzhou 510275, China; 2.广州宏度精细化工有限公司, 广东 广州 510405; 2. Guangzhou Hongdu Fine Chemical Co., Ltd., Guangzhou 510405, China; 3.中山大学应用化学系, 广东 广州 510006; 3. Department of Applied Chemistry, Sun Yat-sen University, Guangzhou 510006, China

**Keywords:** 高效液相色谱, *N*-月桂酰氨基酸, 表面活性剂, 洗面奶, high performance liquid chromatography (HPLC), *N*-lauryl amino acid (NLAAs), surfactants, facial cleanser

## Abstract

*N*-月桂酰氨基酸(NLAAs)表面活性剂与传统表面活性剂相比在安全性上具有明显的优势,应用领域日益增多。但由于NLAAs的紫外吸收较弱,采用高效液相色谱-紫外检测进行含量测定存在一定困难,一般使用示差折光指数检测器、蒸发光散射检测器等通用型检测器来进行检测。为解决这一难题,本研究通过优化流动相的组成,选择合适的色谱柱和检测波长,建立了高效液相色谱-紫外检测同时测定月桂酰谷氨酸(LG)、月桂酰甘氨酸(LC)、月桂酰丙氨酸(LA)、月桂酰肌氨酸(LS)4种NLAAs表面活性剂的方法。样品在乙腈-0.10% H_3_PO_4_水溶液(60∶40, v/v)中超声提取10 min,滤膜过滤后用高效液相色谱仪测定,以乙腈-0.10% H_3_PO_4_水溶液为流动相进行梯度洗脱,经Agilent Eclipse Plus C18色谱柱(150 mm×4.6 mm, 5 μm)分离,在205 nm波长处进行检测。结果显示:4种NLAAs在2.0~800.0 mg/L范围内呈良好的线性关系,相关系数(*r*)均≥0.9995,检出限(LOD)为0.17~0.49 mg/L,定量限(LOQ)为0.57~1.63 mg/L。在0.60、4.50、15.00、24.00 mg/g4个加标水平下,NLAAs的加标回收率在94.3%~107.4%范围内。采用该方法对5个洗面奶样品中的NLAAs进行含量测定,发现所有样品都含有一种或多种NLAAs化合物,NLAAs的总含量在64.58~97.01 mg/g范围内。该方法前处理简单,测试快速,方法精密度、准确性、稳定性良好,适于洗面奶中NLAAs的含量测定,也可为该类型表面活性剂原料纯度、合成产率检测及产品的质量控制提供有效的技术参考。

## Abstract (en)

Significant developments have recently been achieved in the field of *N*-lauryl amino acid (NLAA) surfactants derived from renewable resources. Compared with conventional surfactants, NLAAs exhibit remarkable surfactant properties, exceptional biodegradability, good biocompatibility, and high safety profiles. These attributes have led to the widespread use of NLAAs in personal-care products. The detection methods employed for NLAAs include two-phase titration (TT), spectrophotometric analysis (SA), and high performance liquid chromatography (HPLC). However, because both TT and SA measure the total concentration of anionic active matter, identifying and quantifying individual compounds in a sample containing multiple anionic surfactants is impossible. The presence of cationic surfactants in the sample also introduces interferences, which lead to significant errors. Compared with TT and SA, HPLC offers direct and rapid testing procedures. However, compounds with no or weak UV-visible light absorption exhibit low sensitivity when detected by UV, necessitating the use of detectors such as differential refractive index detectors (RIDs), evaporative light scattering detectors (ELSDs), or charged aerosol detectors (CADs). Most HPLC users consider UV light as the fundamental configuration of the instrument, and other detectors are less commonly employed. Therefore, establishing a new HPLC method suitable for the UV detection of NLAAs is of practical significance.

In this study, a novel HPLC-UV method was developed for the simultaneous detection of *N*-lauryl glutamine (LG), *N*-lauryl glycine (LC), *N*-lauryl alanine (LA), and *N*-lauryl sarcosine (LS) by optimizing the mobile-phase composition and selecting an appropriate chromatographic column and detection wavelength. The samples were mixed with acetonitrile-0.10% H_3_PO_4_ aqueous solution (60∶40, v/v) and sonicated for 10 min, then stayed at room temperature for 5 min. Subsequently, the mixture was filtered through a 0.22 μm filter membrane and separated on an Agilent Eclipse Plus C18 column (150 mm×4.6 mm, 5 μm). The mobile phase used for separation consisted of acetonitrile-0.10% H_3_PO_4_ aqueous solution at a flow rate of 1.0 mL/min. The detection wavelength was set at 205 nm, and the injection volume was 10 μL. The results demonstrated that the four NLAAs exhibited good linearity in the range of 2.0-800.0 mg/L, with correlation coefficients (*r*)≥0.9995. The limits of detection (LODs) ranged from 0.17 to 0.49 mg/L, and limits of quantification (LOQs) ranged from 0.57 to 1.63 mg/L. The relative standard deviations (RSDs) for precision, repeatability, and stability over 24 h were all below 2.0%. Using this method, the NLAA contents of five facial-cleanser products were determined. The results demonstrated that all five samples contained one or more NLAAs, and the total NLAA contents ranged from 64.58 to 97.01 mg/g. The five spiked-sample recoveries of the NLAAs at four different spiked levels (0.60, 4.50, 15.00, 24.00 mg/g) ranged from 94.3% to 107.4%, indicating satisfactory accuracy. However, the actual NLAA composition and label for one facial-cleanser product were not consistent with our test results. This finding demonstrates the necessity of strengthening market monitoring through testing. The proposed method has the advantages of simple pretreatment, rapid testing, good precision, high accuracy, and appropriate stability. Thus, it is suitable for the determination of NLAA contents in facial cleansers and provides an effective technical reference for the raw-material purity assessment, synthetic yield detection, and product quality control of this type of surfactant.

*N*-酰基氨基酸是近年来发展较快、生产合成工艺研究较多、市场占有率不断提升的一类重要表面活性剂^[1⇓⇓-4]^。其合成原料来源于生物质直链脂肪酸和氨基酸,合成时将结构不同的氨基酸和脂肪酸进行组配,得到结构和性能各异的表面活性剂,其中*N*-月桂酰氨基酸(NLAAs)及其盐最为常见。与传统表面活性剂相比,NLAAs性质温和,具有良好的乳化、润湿、增溶、分散、发泡等性能,同时可以降低传统表面活性剂潜在的危险性,表现出优秀的生物降解性、生物相容性和高安全性,现已被广泛应用于日用化学品中^[5⇓⇓⇓⇓⇓⇓-12]^。此外,NLAAs在石油燃料、食品、饮料、机械工业、生物医药、矿物浮选、纺织印染、农药生产等工业领域也都显示出广泛的应用前景^[13⇓⇓-16]^。相关产品由于绿色环保、性能温和的特点,受到人们的欢迎,市场需求日益增大,宣称“温和、安全”的产品也越来越多。相关产品的售价也比传统的表面活性剂高,市场上容易出现以次充好的现象。因此,原料的纯度高低、原料的真假及产品质量好坏的鉴别显得尤为重要。这些问题的存在对NLAAs检测的准确性、便捷性提出了更高的要求。

NLAAs是酰化的羧酸类化合物,属于阴离子表面活性剂。检测阴离子表面活性剂的方法主要有两相滴定法(TT)、亚甲蓝分光光度法(MBSM)、流动注射-亚甲蓝分光光度法(FIA)和高效液相色谱法(HPLC)等^[17⇓-19]^。TT只能测定阴离子表面活性剂的总量,对于多种表面活性剂配伍的样品无法辨别种类并分别定量。对于含有甜菜碱、氧化胺等两性表面活性剂的复配体系,也不能使用两相滴定法,因为这些两性表面活性剂在酸性条件下呈现阳离子性质,会影响阴离子表面活性剂的准确定量^[20]^。此外,用TT对NLAAs进行滴定分析时,容易出现结晶析出或者乳化现象,破乳困难,对滴定的终点判断产生严重干扰^[21]^。MBSM和FIA都是基于阴离子表面活性剂与阳离子染料亚甲蓝反应生成的亚甲基蓝活性物质在特征波长下有吸收的原理进行测试。方法测定的也都是阴离子活性物总量,而且样品中的阳离子活性物、硫酸盐、磺酸盐及酚类等配伍化合物都会对测试有干扰,导致结果偏高或偏低,必须要先消除干扰物才能进行测试。这两种方法主要被用于水质中低浓度的阴离子表面活性剂的测定,对于洗护产品这类成分复杂且含有高浓度表面活性剂的样品并不适合。与传统方法相比,HPLC具有测试直观、快速的优点,但从现有文献^[22⇓-24]^来看,该法一般是使用紫外检测器(UV)或荧光检测器检测带有芳香环结构或有较强紫外吸收的表面活性剂,如直链烷基苯磺酸盐(LAS)等。对于无或弱紫外吸收的化合物用UV检测灵敏度低,一般采用示差折光指数检测器(RID)、蒸发光散射检测器(ELSD)或电雾式检测器(CAD)^[25⇓⇓-28]^等通用型检测器。对于大多数高效液相色谱仪用户来说,基本上80%以上的化合物都是用UV来进行测试,UV是HPLC仪器的基本配置,其他3种检测器则较少配置。因此,建立HPLC-UV新方法对NLAAs的测试具有更高的应用价值。

洗护产品如洗面奶基质复杂,通常含有一种或多种NLAAs,目前市场上较常使用的NLAAs有月桂酰谷氨酸(LG)、月桂酰甘氨酸(LC)、月桂酰丙氨酸(LA)及月桂酰肌氨酸(LS),若能同时检测这4种化合物则可以极大地提高检测效率。本研究针对这4种化合物的弱紫外吸收特点,通过筛选合适的流动相,选择检测波长及色谱柱,建立了HPLC-UV同时测定洗面奶中4种NLAAs含量的方法,为NLAAs原料的纯度分析,原料生产监控及洗护产品的品质控制提供了新的技术支撑。

## 1 实验部分

### 1.1 仪器与试剂

Agilent 1290超高效液相色谱仪(美国安捷伦公司); XS205Du电子天平(美国梅特勒托利多公司); CPX5800H-C超声波清洗仪(美国Branson公司); Milli-Q超纯水机(美国Millipore公司)。甲醇、乙腈(色谱纯,美国Thermo公司);磷酸(分析纯,广州化学试剂厂); LG、LC、LA和LS对照品(纯度≥98%,广州宏度精细化工有限公司)。

### 1.2 对照品溶液的配制

分别精密称取约8 mg(精确至0.01 mg)LG、LC、LA及LS对照品于4个10 mL容量瓶中,加入8 mL乙腈-0.10%H_3_PO_4_水溶液(60∶40, v/v),超声溶解10 min,静置至室温,用乙腈-0.10%H_3_PO_4_水溶液(60∶40, v/v)定容,得到质量浓度为800.0 mg/L的4种NLAAs对照品储备溶液。配制时用乙腈-0.10%H_3_PO_4_水溶液(60∶40, v/v)逐级稀释,配成质量浓度为2.0、10.0、20.0、50.0、100.0、200.0、400.0、560.0、800.0 mg/L的系列对照品工作溶液。

### 1.3 样品前处理

取洗面奶样品大约10 mL,涡旋分散均匀后,准确称取约80 mg(精确至0.01 mg)样品至10 mL容量瓶中,加入约8 mL乙腈-0.10% H_3_PO_4_水溶液(60∶40, v/v),超声提取10 min后,静置至室温,用乙腈-0.10% H_3_PO_4_水溶液(60∶40, v/v)定容至刻度线,混匀,放置5 min,取适量上清液经0.22 μm有机滤膜过滤,滤液供高效液相色谱仪上机测试(供试品溶液可根据实际浓度用乙腈-0.10% H_3_PO_4_水溶液(60∶40, v/v)进行适当稀释)。

### 1.4 液相色谱条件

色谱柱:Agilent Eclipse Plus C18色谱柱(150 mm×4.6 mm, 5 μm);柱温:35 ℃;进样量:10 μL;流速:1 mL/min;流动相A: 0.10% H_3_PO_4_水溶液;流动相B:乙腈。检测波长:205 nm。梯度洗脱条件:0~2.0 min, 60%B; 2.0~8.0 min, 60%B~95%B; 8.0~10.0 min, 95%B; 10.0~10.1 min, 95%B~60%B; 10.1~15.0 min, 60%B。

## 2 结果与讨论

### 2.1 实验条件考察

#### 2.1.1 提取溶剂选择

*N*-月桂酰氨基酸表面活性剂既有疏水基团又有亲水基团,在有机溶剂和水溶液中均有较好的溶解性。参考已有标准及文献^[25⇓⇓-28]^,目前洗护产品中*N*-月桂酰氨基酸的提取主要是使用甲醇、乙腈、水、甲醇-水或乙腈-水的直接提取法,此法具有步骤简单、操作便捷的特点。根据卓文珊等^[26]^对表面活性剂原料使用不同比例的乙腈-水溶液提取月桂酰甘氨酸的研究结果,60%乙腈水溶液与80%乙腈水溶液的提取效果较好而且两者之间差异非常小,为减少色谱分析过程中溶剂效应的影响,本研究选择色谱条件中的初始流动相即乙腈-0.10% H_3_PO_4_水溶液(60∶40, v/v)作为提取溶剂。

#### 2.1.2 检测波长选择

LG、LC、LA和LS的结构式见图1。4种NLAAs的结构比较相似,分子结构中的C=O不饱和键含有未共用单子对,能产生*n*-*π*跃迁,由于跃迁能量较低,紫外吸收一般比较弱。对4种化合物的紫外吸收光谱图进行分析,发现当波长低于240 nm时才开始有微弱的紫外吸收,吸收强度随着波长的变短而增强,最大吸收波长约在205 nm处,因此方法选择205 nm作为检测波长。

**图1:**
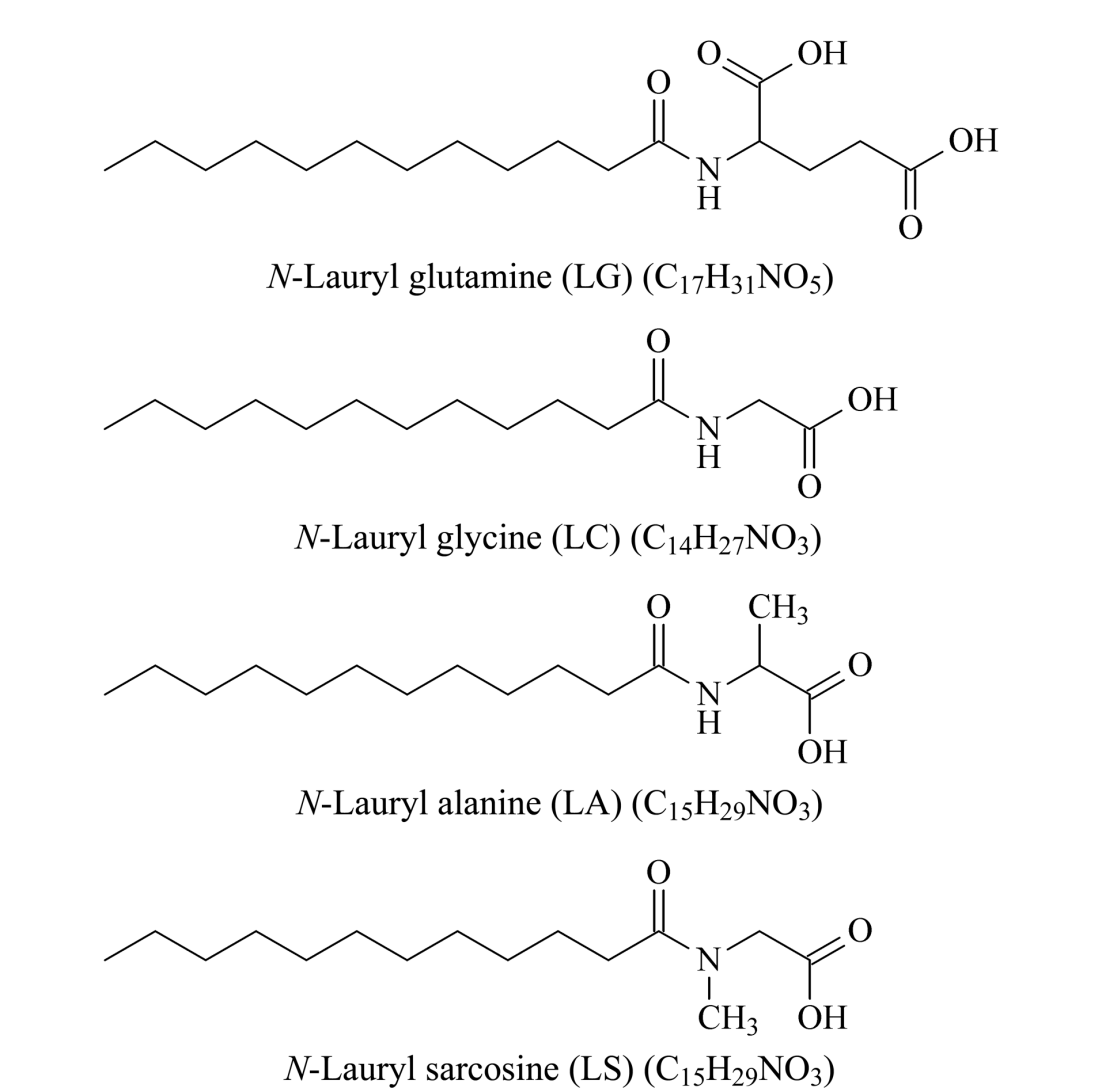
LG、LC、LA及LS的化学结构式

#### 2.1.3 酸性添加剂选择

4种NLAAs结构中都含有1个或2个羧基,结构与有机酸类似,一般首选酸性缓冲溶液作为水相。但大多数酸性添加剂在205 nm波长下也都有吸收,容易带来干扰,因此必须进行筛选。选择HPLC常用的3种酸性添加剂包括1种无机酸(磷酸)及2种有机酸(乙酸和三氟乙酸),考察基线波动及基线漂移的情况。结果显示,采用磷酸时的基线比采用乙酸和三氟乙酸溶液时的基线更稳定,梯度洗脱时对基线漂移的影响也更小。因此本方法选择磷酸作为酸性添加剂。

#### 2.1.4 流动相酸度选择

分别添加0.05%、0.075%、0.10%、0.125%和0.15%磷酸到水相中,运行相同的洗脱程序,考察酸度变化对4种NLAAs峰面积和保留时间的影响,色谱图见图2。结果显示,酸度变化对NLAAs保留时间的影响不大,基本保持不变。但随着磷酸体积分数的增加,峰形有所改善,峰的前延伸现象消失,分离度稍微增大。由于LG是双羧基结构,添加0.05%磷酸时,由于酸度不够,部分羧基解离,出现两个色谱峰。添加0.075%磷酸时,LG仍可以看到肩峰现象,当磷酸≥0.10%后,酸度的增强完全抑制了LG羧基的解离,只有一个峰,且随着酸度的继续增大峰面积基本保持不变。

**图2:**
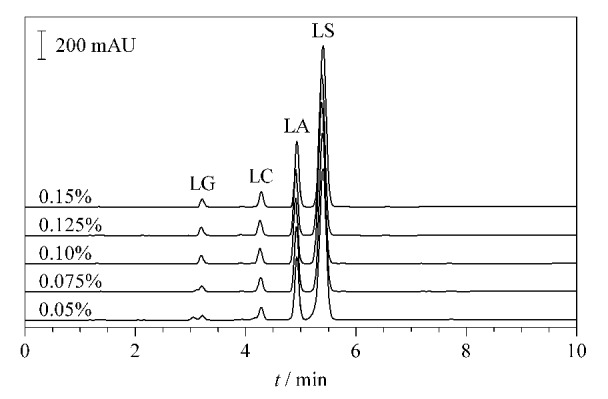
磷酸体积分数对LG、LC、LA和LS峰面积与保留时间的影响

其他3种化合物是单羧基结构,磷酸的体积分数对峰面积和保留时间的影响不大,基本上保持不变。LC和LS的色谱峰对称性随着酸度的增大而有所改善,在磷酸≥0.10%后基本保持不变。考虑到流动相的酸性太强对色谱柱会有一定损害,而实际样品的酸碱度也会有差异,有可能是弱碱性,流动相须具备一定的缓冲能力,因此选择磷酸的体积分数为0.10%。

#### 2.1.5 洗脱方式及有机相选择

洗面奶产品的配方成分通常都比较复杂,可能含有脂肪酸(如棕榈酸、硬脂酸等)合成的强保留表面活性剂,使用梯度洗脱比等度洗脱更为合适。本文首先考察了使用甲醇和0.10% H_3_PO_4_水溶液作为流动相的情况,比较了运行含60%甲醇流动相的等度洗脱程序和1.4节梯度洗脱程序对样品分离效果的差异。结果显示,梯度洗脱没有出现等度洗脱时色谱峰轻微拖尾的现象,可以得到更加对称的色谱峰,而且样品中的强保留组分也快速流出,因此选择梯度洗脱。然后考察了甲醇、乙腈作为有机相的差别,使用甲醇时基线出现了较严重的波动与漂移现象,不利于低浓度目标化合物的检测。乙腈的截止波长比甲醇更低,在同样的梯度洗脱程序下,乙腈比甲醇的基线波动和基线漂移更低,因此选择乙腈作为有机相。

#### 2.1.6 色谱柱选择

NLAAs是一类既有长链脂肪烃,又有羧基的阴离子表面活性剂。长链脂肪烃对色谱柱产生较强的吸附作用,羧基则容易产生次级保留作用,容易导致色谱峰拖尾。实验考察了不同类型的C18色谱柱,发现经过封端处理的Agilent Eclipse Plus C18色谱柱效果理想,因此选择Agilent Eclipse Plus C18色谱柱来进行方法验证。按照1.4节方法进行测试,4种NLAAs对照品的色谱图见图3a,此时4种化合物的色谱峰峰形尖锐,对称性良好,理论塔板数在9700~20000范围内,拖尾因子为0.93~0.98。洗面奶加标样品的色谱图见图3b,可以看到4种目标化合物与洗面奶样品中的其他成分均达到理想分离,无杂质干扰,满足分析要求。

**图3:**
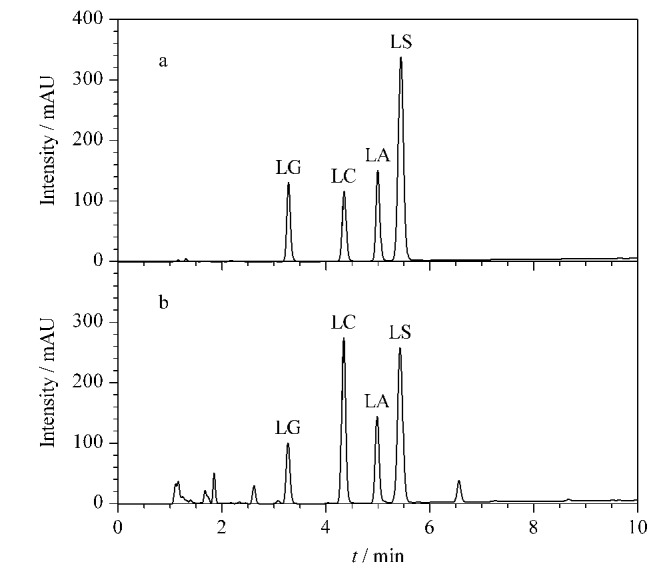
(a)对照品及(b)加标样品的色谱图

### 2.2 方法学考察

#### 2.2.1 线性关系、检出限和定量限

取配制好的4种化合物系列对照品溶液,按1.4节色谱条件进行分析。以对照品质量浓度*X*(mg/L)为横坐标,峰面积*Y*为纵坐标进行线性回归,分别以信噪比(*S/N*)为3和10时的浓度作为分析方法的检出限(LOD)和定量限(LOQ),结果见表1。结果显示:4种NLAAs在2.0~800.0 mg/L范围内都具有良好的线性关系,*r*≥0.9995。方法的LOD较低,为0.17~0.49 mg/L, LOQ为0.57~1.63 mg/L。

**表1:** 4种NLAAs的线性范围、线性方程、相关系数、检出限和定量限

Compound	Linear range/(mg/L)	Linear equation	r	LOD/(mg/L)	LOQ/(mg/L)
LG	2.0-800.0	Y=3.23778717X+5.4264289	0.9996	0.42	1.40
LC	2.0-800.0	Y=3.0579659X+3.0810632	0.9995	0.49	1.63
LA	2.0-800.0	Y=3.83648389X+5.4939317	0.9997	0.38	1.27
LS	2.0-800.0	Y=12.1006233X+5.2556411	0.9997	0.17	0.57

*Y*: peak area; *X*: mass concentration, mg/L.

#### 2.2.2 仪器精密度和对照品稳定性

取质量浓度为50.0和200.0 mg/L的4种NLAAs对照品溶液,进行6次平行测定,4种目标化合物保留时间的RSD值为0.04%~0.23%,含量的RSD值为0.02%~0.24%。取上述溶液置于室温下,于制备后0、12和24 h进样测定,得到对照品稳定性结果,24 h内4种NLAAs含量的RSD值为0.28%~0.84%。表明仪器的重复性良好,对照品溶液在24 h内稳定性好。

### 2.3 与已有报道方法的比较

与TT、MBSM等传统方法相比,HPLC方法可以更加精准地得到氨基酸表面活性剂的具体含量,所用试剂种类少,而且无需使用三氯甲烷等有毒溶剂,降低了有机溶剂的消耗和环境污染问题。本方法使用UV检测器,UV检测器与ELSD、RID、CAD等检测器相比,具有适用性广、检出限低、线性范围宽的优点。以检出限为例,本方法对LG、LC、LS的检出限分别为0.42、0.49、0.17 mg/L,而已有报道的HPLC-ELSD对LC的检出限为3.0 mg/L^[26]^,对LG的检出限为50 mg/L^[25]^, HPLC-CAD对LS的检出限为3.75 mg/L^[28]^。由此可见本方法的检出限远低于已有的文献方法。此外,本方法操作简单,耗时短,HPLC对每个样品中4种NLAAs的单针检测过程在15 min内就能完成,极大地提升了样品的检测效率。而且方法使用的UV检测器是最通用的检测器,也有利于方法的产业化推广使用。

### 2.4 样品分析

采用本方法对网上售卖的来源于不同厂家的5种氨基酸型洗面奶产品(编号:S1~S5)进行NLAAs含量检测,结果见表2。5个样品均含有一种或多种NLAAs, NLAAs的总含量在64.58~97.01 mg/g范围内。所有样品都含有LC, 含量为59.25 ~75.55 mg/g。此外,洗面奶S1、S2和S5中还含有LG,含量为2.55~37.76 mg/g,洗面奶S4中含有LA,含量为9.31 mg/g。将检测结果与产品标签进行比较发现:样品S1~S4含有的NLAAs成分与产品标签保持一致,但S5中并没有检测到标签中标有的主要成分LS,实际检测到的是未在标签中列出的LC。结果表明对此类氨基酸表面活性剂产品进行监管十分必要。采用本法在5个洗面奶样品中精密加入不同体积的4种NLAAs对照品溶液,进行加标回收试验,结果见表2。LG、LC、LA和LS在4个水平下的加标回收率为94.3%~107.4%。可见方法的回收率较高,准确度良好。使用此方法可以准确地检测4种NLAAs的含量,为洗面奶产品监管提供技术支撑。

**表2:** 实际样品中4种NLAAs的含量和加标回收率

Compound	Added/ (mg/g)		S1						S2						S3						S4						S5					
			Found/ (mg/g)		Recovery/ %				Found/ (mg/g)		Recovery/ %				Found/ (mg/g)		Recovery/ %				Found/ (mg/g)		Recovery/ %				Found/ (mg/g)		Recovery/ %	
LG	0		2.55		/				25.44		/				-		/				-		/		37.76					/
	0.60		3.09		98.0				26.01		99.9				0.57		95.3				0.59		99.0		38.32					99.9
	4.50		7.21		102.2				30.78		102.8				4.83		102.0				4.83		107.3		43.02					101.8
	15.00		18.27		104.1				41.33		102.2				15.54		103.6				15.86		105.7		54.71					103.7
	24.00		26.02		98.0				48.75		98.6				23.09		96.2				23.71		98.8		61.76					100.0
LC	0		62.03		/				62.39		/				68.09		/				75.55		/		59.25					/
	0.60		62.63		100.0				62.93		99.9				68.83		100.2				76.30		100.2		59.91					100.1
	4.50		68.13		102.4				68.56		102.5				74.40		102.5				80.85		101.0		64.90					101.8
	15.00		78.88		102.4				77.93		100.7				91.28		102.1				91.09		100.6		76.55					103.1
	24.00		85.60		99.5				84.83		98.2				96.24		97.8				96.76		97.2		83.17					99.9
LA	0		-		/				-		/				-		/				9.31		/		-					/
	0.60		0.64		107.3				0.64		106.3				0.63		104.5				9.92		100.1		0.62					103.8
	4.50		4.62		102.7				4.41		98.0				4.55		101.0				14.64		106.0		4.68					104.0
	15.00		16.10		107.3				16.11		107.4				16.11		107.4				25.65		105.5		15.72					104.8
	24.00		23.83		99.3				23.93		99.7				23.69		98.7				33.08		99.3		23.30					97.1
LS	0		-		/				-		/				-		/				-		/		-					/
	0.60		0.60		99.8				0.60		100.3				0.60		99.5				0.61		101.8		0.62					103.8
	4.50		4.42		98.3				4.40		97.7				4.44		98.7				4.41		98.0		4.46					99.0
	15.00		15.29		101.9				15.27		101.8				15.33		102.2				15.17		101.1		15.20					101.3
	24.00		22.99		95.8				22.87		95.3				22.75		94.8				22.82		95.1		22.63					94.3

-: not detected; /: no data.

## 3 结论

本研究通过优化流动相、选择合适的色谱柱和检测波长,解决了弱紫外吸收表面活性剂的检测难题,建立了HPLC-UV同时检测LG、LC、LA和LS 4种NLAAs的方法。使用本方法对市售的5款洗面奶进行氨基酸表面活性剂的含量检测,结果发现其中1款洗面奶含有的NLAAs类型与产品标签不吻合,证明了对相关产品进行检测的必要性。方法的前处理操作简单,精密度、准确性、稳定性良好,为洗面奶产品中NLAAs的分析提供了可靠的技术手段,在*N*-月桂酰氨基酸表面活性剂的原料生产监控、化妆品质量控制以及化妆品市场监控等方面均具有较强的实用价值。
